# Correction: Prevalence and risk factors for hepatitis C virus infection in an informal settlement in Karachi, Pakistan

**DOI:** 10.1371/journal.pgph.0002897

**Published:** 2024-01-29

**Authors:** Munazza Mansoor, William A. de Glanville, Ridwa Alam, Khawar Aslam, Mubashir Ahmed, Petros Isaakidis, Aneeta Pasha

There are errors in the Funding and Competing Interests statements. The correct statements are:

**Funding:** This Project was funded by Foundation for Innovative New Diagnostics, which had no role in the study design, data collection and analysis, decision to publish, or preparation of the manuscript. Médecins Sans Frontières (MSF) provided support for the study in the form of salaries for WAdG, KA, MA, and PI. The specific roles of these authors are articulated in the ‘author contributions’ section.

**Competing Interests:** The authors have read the journal’s policy and have the following competing interests to declare: MSF provided support for the study in the form of salaries for WAdG, KA, MA, and PI. This does not alter our adherence to *PLOS Global Public Health* policies on sharing data and materials. There are no patents, products in development or marketed products associated with this research to declare.
