# Supplementary material for: Prevalence and risk factors for hepatitis C virus infection in an informal settlement in Karachi, Pakistan
Source: PLOS Glob Public Health. 2023 Sep 20;3(9):e0002076. doi: 10.1371/journal.pgph.0002076 (PMC10511086; doi:10.1371/journal.pgph.0002076)
Supplement: S2 File — (RTF) [file pgph.0002076.s002.rtf]

Supplementary Materials
Manuscript Title: Prevalence and risk factors for hepatitis C virus infection in an informal settlement in Karachi, Pakistan
ID: PGPH-D-23-00220

grid_no _anonymised = grid cell in which household falls (anonymised)
rdt_result = result of rapid diagnostic test (0 = negative; 1 = reactive)
pcr_res = result of confirmatory PCR result (0 = HCV not detected; 1 = HCV detected)
.gender = gender (1 = female; 0 = male)
age = reported age in years
.transfusion = received transfusion in lifetime (1 = yes; 0 = no)
.fishing = reported to work in fishing sector (fisherman or fish/shrimp processing) (1 = yes; 0 = no)                    
.injection = received at least one injection in past 12 months 
.injection_num2 = number of injections received in past 12 months
.dentist = reported ever used dentist               
.hcv_knowledge = generated variable for HCV awareness (see main text) (1 = HCV aware; 0 = HCV unaware)
Responses to the question: “Do you know any ways a person can avoid getting Hepatitis C”
hcv_prevent_razors = Avoid sharing used razor     
hcv_prevent_contact = Avoid contact with infected persons  
hcv_prevent_nosay = Don't know/Prefer not to say
hcv_prevent_baby = Have a baby in a hospital   
hcv_prevent_mossie  = Avoid mosquito bites   
hcv_prevent_toothbrush = Avoid sharing toothbrushes   
hcv_prevent_injections = Avoid unsterilized syringes and medical equipment  
hcv_prevent_contactskin = Avoid contact with damaged skin 
hcv_prevent_bodyfluids  = Avoid contact with infected bodily fluids 
hcv_prevent_safesex = Practice safe sex   
hcv_prevent_safeblood = Safe blood transfer     
hcv_prevent_disposablesyringe = Use disposable syringe
hcv_prevent_peros = Avoid contaminated food/water  
hcv_prevent_other1s = Other   
