# Supplementary material for: Prevalence and risk factors for hepatitis C virus infection in an informal settlement in Karachi, Pakistan
Source: PLOS Glob Public Health. 2023 Sep 20;3(9):e0002076. doi: 10.1371/journal.pgph.0002076 (PMC10511086; doi:10.1371/journal.pgph.0002076)
Supplement: S3 File — (PDF) [file pgph.0002076.s003.pdf]

IRD Pakistan

Hepatitis C (HCV) Prevalence Survey

Table of Contents

Part 1: Instructions to the Surveyor **2**
- Time Frame 2
- Introduce the Survey 2
- Administer the Consent 2
- Facilitating the Survey 3
- Ending the Survey 3

Part 2: Survey **4**
- Questionnaire 01: Household Information 4
- Questionnaire 02: Individual Information 9
- Questionnaire 03: HCV-Specific Information 15

<u>Part 1: Instructions to the Surveyor</u>

Purpose: The Hepatitis C Prevalence Survey will be administered to a random selection of participants who reside in Machar Colony. The aim of this survey is to examine the prevalence of exposure and infection with the hepatitis C virus (HCV) within the said locale.

<u>Time Frame</u>

Screening will be conducted within 434 households of Machar Colony, with each taking approximately 15-20 minutes.

<u>Introduce the Survey</u>

Instructions

Before administering the survey, start by introducing yourself. Acknowledge the participation of the household member and give them a brief overview of the survey being conducted. The surveyor should inform the participant that the questionnaire will follow a structured format.

As-salamalaikum, my name is [identify self] and I am a part of a research team for the Hepatitis C Prevalence Survey from the Interactive Research and Development (IRD). I want to thank you for taking the time to speak with me today. Today, as one of the components of the survey, I would like to speak with you in order to gain an understanding of how common HCV is within Machar Colony and who is most at risk of infection. The screening should take 15-20 minutes approximately. If you provide us with your consent, as part of this screening, we will ask you questions and administer a test for HCV.

If the participant agrees to participate, acquire their consent and administer the subsequent three questionnaires (Household Questionnaire, Individual Questionnaire and the HCV-specific Questionnaire). If they refuse to participate, ask them if they would be willing to answer a short questionnaire (Household Questionnaire for households who decline any HCV testing and Individual Questionnaire for individuals in participating households who decline HCV testing) without testing. If they agree, administer the Household Questionnaire and the Individual Questionnaire only. If they refuse, no data is to be collected from the specified household.

<u>Administer the Consent</u>

Instructions

Provide a brief outline of the purpose of the prevalence survey, the kind of information needed from the participants, and how the information will be used by IRD and MSF.

Allow the participant time to read or read out the informed consent form ("HCV Consent Form") to all participants older than 18 years of age and record their responses. Similarly, administer consent form ("HCV Guardian Consent Form") to legal guardians so that they may provide consent for minors from within the age bracket of 12-17 years. Upon acquiring consent from the

guardian, gain assent from the minor by administering the informed assent form ("HCV Assent Form"). For those choosing not to be a part testing, seek consent for collecting information on the questionnaire. For adults, administer consent form ("HCV Questionnaire Consent Form"). Similarly, for all minors, administer consent to the guardian first ("HCV Questionnaire Guardian Consent Form") and subsequently, to the minor ("HCV Questionnaire Assent"). For those who are not able to provide written consent, obtain a thumb impression. Whilst administering assent to a minor, their legal guardian must be present in the room at all times.

All participants should be provided an opportunity to ask as many questions as they would like regarding their participation in the survey. Ensure all those who agree to participate, that their contact information and responses will be kept confidential, pseudonymized through datasets and not be used for any other purpose.

Facilitating the Survey

1. It is very important to build a rapport with the participants and make them feel comfortable. Try to maintain a controlled tone of voice and remain neutral to encourage open participation.
2. If the participant is not providing much information and is reserved, request for them to share their opinion in an attempt to encourage participation.
3. In case of a difficult situation, for example a sensitive topic which causes the participant to be uneasy, you can move onto the next question. You may come back to it later if the participant feels relaxed and agrees to answer the question. If the participant does not agree to answer any question, you can mark it as 'Prefer not to say'. Throughout the process, the surveyor must ensure that they are as comfortable as possible so that the participant may also be put at ease.
4. Do not judge any responses received. The surveyor must try to collect information as objectively as possible.
5. Based upon the result of the participant in Questionnaire 03 (HCV-Specific Questionnaire), create a referral slip and link them to the MSF clinic for free-of-cost testing and further treatment.

Ending the Survey

When the survey is ending, thank the household members for their participation and address all questions or concerns of the participant. Provide contact details of the Assistant Program Manager, in case they have any questions.

Contact Details:
Name: Munazza Mansoor
Contact: + 92 349 2052275

Part 2: Survey

Try to ask all the questions below in the order given in the three-part survey. More importantly, try to maintain a smooth flow of discussion during this process and use the skip logic where applicable. The household questionnaire can be asked of one person in the household. This should be an adult (>18 years) with sufficient knowledge about the household.

If the participant has consented to the research activity, administer all 03 questionnaires and conduct the RDT accordingly. However, as mentioned previously, if they refuse, seek their consent on whether you can collect data on the first 02 questionnaires.

Questionnaire 01: Household Information

No.	Question	Response Code
1	Screening Date	_ _ / _ _ / _ _ _ _ (DD/MM/YYYY)
2	Grid cell number	
3	Surveyor ID	
4	Household ID	IRD_HH_ _ _ _ _ _ _ _ _ _ _
5	How many people in the age categories live (sleep) in the same house with you at the moment?<br><br>*(Enumerator: we are defining a household as a single dwelling in which a group of people live together. It includes people who normally stay and those who stayed last night)*	• Elderly (65+):_______________<br>• Adults (18-64):_____________<br>• Children (6-17):_____________<br>• Children (under 6):___________<br>• Prefer not to say
6	What type of fuel do you mainly use for cooking?<br><br>*(Select main source mentioned)*	• Electricity<br>• LPG<br>• Natural gas<br>• Kerosene

		- Charcoal - Wood - No food cooked in household - Other (please specify) - Prefer not to say
7	Does anyone in this household own any of the following?<br><br>*(Read each to respondent)*	- A watch - A mobile phone - A bicycle - A motorcycle or motor scooter - An animal drawn cart - A car or truck or bus - A boat with a motor - A boat without a motor - A fishing net - A rickshaw/chingi - Prefer not to say
8	What is the main source of drinking water for members of your household?<br><br>*(Select main source mentioned)*	- Piped into dwelling - Piped into yard/plot - Piped to neighbour - Public tap - Water through tankers - Cart with small tank - Bottled water - Borehole (tube well) - Protected well - Unprotected well - Rainwater - Surface water (spring/pond/river/stream water/canal) - Prefer not to say
9	What kind of toilet facility do members of your household usually use?<br><br>*(Select main source mentioned)*	- Flush toilets to a piped sewer - Flushed to somewhere else - Ventilated improved pit latrine - Pit latrine with slab - Pit latrine without slab - Bucket toilet

		• No facility<br>• Other (please specify)<br>• Prefer not to say
10	Including this household, how many households use this toilet facility?	• Number if less than 10:______<br>• 10 or more<br>• Do not know<br>• Prefer not to say
11	*Enumerator*: Observe and record main material of the floor of the dwelling	• Earth/sand<br>• Wood planks<br>• Palm/bamboo<br>• Polished wood<br>• Vinyl<br>• Tiles<br>• Cement<br>• Bricks<br>• Carpet/mats<br>• Other (please specify)
11.1	*Enumerator*: Observe and record main material of the roof of the dwelling.	• No roof<br>• Plastic<br>• Thatch/palm<br>• Mud<br>• Wood planks<br>• Cardboard<br>• Cement<br>• Metal<br>• Tiles<br>• Other (please specify)
11.2	*Enumerator*: Observe and record main material of the exterior walls of the dwelling.	• No walls<br>• Plastic<br>• Thatch/palm<br>• Mud stones<br>• Unbaked bricks<br>• Wooden planks<br>• Reused wood

		• Cement<br>• Cement blocks<br>• Bricks<br>• Other (Please specify)

IRD Pakistan

Hepatitis C (HCV) Prevalence Survey

Individual Questionnaire

Questionnaire 02: Individual Information

No.	Question	Response Code
**Section A: Individual Information**		
1	Household ID	IRD_HH_ _ _ _ _ _ _ _ _ _ _
2	Individual ID	IRD_ _ _ _ _ _ _ _ _ _ _ _ _
3	Age (in years)	_ _
3.1	Age group (if age in years not known)	● 12-17<br>● 18-29<br>● 30-39<br>● 40-49<br>● 50-59<br>● ≥60
4	Gender	● Female<br>● Male<br>● Other
5	Do you normally stay in this household?<br>*By "normally", we mean is this the place you spend most of your nights or plan to spend most nights in the future.*	● Yes → Q 6<br>● No → Q 5.1<br>● Prefer not to say
5.1	Do you normally stay in Machar Colony?<br>*By "normally", we mean is Machar Colony the place you spend most of your nights or plan to spend most nights in the future.*	● Yes<br>● No<br>● Prefer not to say

6	What is the highest level of education you have completed?	• No schooling<br>• Some primary school<br>• Primary school<br>• Middle school<br>• Secondary school<br>• Intermediate<br>• Post-secondary certificate<br>• Higher level courses (such as diploma, university)<br>• Prefer not to say
7	Marital status	• Single<br>• Married<br>• Divorced<br>• Widowed<br>• Prefer not to say
8	Have you done any paid work in the past 7 days?	• Yes → Q 8.2<br>• No → Q 8.1<br>• Prefer not to say
8.1	If not, have you done any paid work in the past 12 months?	• Yes<br>• No<br>• Prefer not to say
8.2	How would you describe your employment?	• Part time employed by someone else<br>• Full time employed by someone else<br>• Self-employed<br>• Subsistence<br>• Volunteering<br>• Student<br>• Other (please specify)<br>• Prefer not to say

<table>
<tr><td>8.3</td><td>Do you usually work throughout the year, or do you work seasonally, or only once in a while?</td><td>Throughout the year
Seasonally/part of the year
Once in a while
Prefer not to say</td></tr>
<tr><td>8.4</td><td>What work do you do?

(Record all that are mentioned)</td><td>Worker at fisheries
Fish /shrimps vendor
Rubbish collector
Construction worker
Fisherman
Traditional birth attendant
Businessman
Sailor
Transporter
Driver
I care for my family at home
Self-employed
Government employee
Other (please specify)
Prefer not to say</td></tr>
<tr><th colspan="3">Section B: Communal Knowledge</th></tr>
<tr><td>1</td><td>Do you know any ways a person can avoid getting Hepatitis C?

Record all that are mentioned.</td><td>Avoid sharing used razors
Avoid sharing toothbrushes
Avoid unsterilized syringes and medical equipment
Avoid contact with damaged skin
Avoid contact with infected bodily fluids
Practice safe sex
Safe blood transfer
Use disposable syringe
Avoid contaminated food/water
Avoid contact with infected persons
Have a baby in a hospital
Avoid mosquito bites
Other (please specify)
Prefer not to say</td></tr>
<tr><th colspan="3">Section C: Risk Factors</th></tr>
</table>

2	Have you ever undergone a surgery?<br><br>*By "surgery" we mean a procedure in which a doctor cut into the body*	• Yes<br>• No<br>• Prefer not to say
2.1	If yes, can we ask when the last time was?	• _ _ / _ _ _ _ (MM/YYYY)<br>• Prefer not to say
3	Have you ever had to stay in a hospital or other health facility for treatment overnight?	• Yes<br>• No<br>• Prefer not to say
3.1	If yes, can we ask when the last time was?	• _ _ / _ _ _ _ (MM/YYYY)<br>• Prefer not to say
4	Have you ever received a blood transfusion?	• Yes<br>• No<br>• Prefer not to say
5	Have you had an injection for any reason in the last 12 months?<br><br>*We mean when a needle and syringe is used to put a liquid in the body, or to take a liquid from the body.*	• Yes → Q 5.1<br>• No → Q 6<br>• Prefer not to say
5.1	How many injections have you had in the past 12 months?	• Number of injections: __<br>• Do not know<br>• Prefer not to say
5.2	Among these injections, how many were administered by a doctor, nurse, pharmacist, or any other health worker?<br><br>*Enumerator: make clear these are "formal" health providers.*	• Number of injections: __<br>• None
5.3	The last time you got an injection from a health worker, did he/she take the syringe and needle from a new, unopened package?	• Yes<br>• No<br>• Do not know<br>• Prefer not to say

6	Have you ever visited a dentist and had a procedure done on your teeth?	• Yes → Q 6.1<br>• No<br>• Prefer not to say
6.1	If yes, have you visited a dentist to have a procedure done on your teeth in the past 12 months?	• Yes<br>• No<br>• Prefer not to say
7	Have you ever gotten a piercing?	• Yes<br>• No<br>• Prefer not to say
8	Have you ever had a tattoo?	• Yes<br>• No<br>• Prefer not to say
9	Have you visited a barber in the past 12 months?	• Yes → Q 9.1<br>• No<br>• Prefer not to say
9.1	If yes, have you visited a barber in the past month?	• Yes<br>• No<br>• Prefer not to say
9.2	The last time you visited a barber, did he/she take the blade from a new, unopened packet?	• Yes<br>• No<br>• Do not know<br>• Prefer not to say
10	In the past 12 months, have you directly assisted a woman to give birth in her household?<br><br>*Enumerator: this should include helping women to give birth in this household*	• Yes → Q 10.1<br>• No<br>• Prefer not to say
10.1	If yes, how many women have you assisted to give birth?	• 1<br>• 2 - 4<br>• 5 - 10

		• > 10<br>• Prefer not to say

<u>IRD Pakistan</u>

<u>Hepatitis C (HCV) Prevalence Survey</u>

<u>HCV-Specific Questionnaire</u>

Questionnaire 03: Test Result Information

No.	Question	Response Code
	**Section A: HCV History**	
1	Household ID	IRD_HH_ _ _ _ _ _ _ _ _ _ _
2	Individual ID	IRD_ _ _ _ _ _ _ _ _ _ _ _ _
3	Have you ever been tested for HCV?	• Yes → Q 3.1<br>• No → Q 3.5<br>• Do not know → Q 3.5<br>• Prefer not to say → Q 3.5
3.1	If you have been tested for HCV, what was the date of the last HCV test?	_ _ / _ _ _ _ (MM/YYYY)
3.2	Where was the test done?	• Within the HCV clinic in Machar Colony<br>• During mass screening event in Machar Colony<br>• Outside of Machar Colony in a private facility<br>• Outside of Machar Colony in a public facility<br>• Other (please specify)
3.3	Give name of facility, if known	
3.4	If you have been tested for HCV, would you be willing to tell me the result of your last HCV test?	• Positive<br>• Negative<br>• Did not get results<br>• Do not know<br>• Prefer not to say
3.5	Do you know a place where people	• Facility within Machar Colony

	can go get tested for HCV?	● Facility outside Machar Colony<br>● Private facility<br>● Public facility<br>● Other<br>● No<br>● Prefer not to say
4	Have you ever been treated for HCV by a doctor or other medical professional?	● Yes – currently on treatment<br>● Yes – but didn't complete treatment<br>● Yes – completed treatment<br>● No<br>● Prefer not to say
5	Can we ask if you have been told that you have any of the following conditions?	● Diabetes<br>● HIV infection<br>● HBV infection<br>● Heart disease<br>● Hypertension<br>● Asthma<br>● Chronic obstructive pulmonary disease (COPD)<br>● TB<br>● None of the above<br>● Prefer not to say
**Section B: Screening Outcome**		
6	RDT Result	● Reactive<br>● Non-reactive
7	Does the patient indicate they will go to MSF for further testing?	● Yes<br>● No → Q 7.1
7.1	Reason they are unwilling to go?<br><br>*Select all that apply*<br><br>*Enumerator: Make sure that the participant is aware that further testing is required to confirm their RDT reactive result, and if this is*	● Individual says it will take too long<br>● Individual is too busy<br>● Individual cannot afford time off work<br>● Individual cannot travel to clinic on their own

	positive, they are likely to benefit from free treatment against Hepatitis C at the MSF clinic	● Individual did not feel the need to get tested<br>● Individual is worried about starting treatment<br>● Other (please specify)
