# Supplementary material for: Prevalence and risk factors for hepatitis C virus infection in an informal settlement in Karachi, Pakistan
Source: PLOS Glob Public Health. 2023 Sep 20;3(9):e0002076. doi: 10.1371/journal.pgph.0002076 (PMC10511086; doi:10.1371/journal.pgph.0002076)
Supplement: S4 File — (DOCX) [file pgph.0002076.s004.docx]

**Supplementary Materials**

Below is the R code to perform the logistic regression with seropositivity on the screening test as the outcome, including estimation of missing values in the predictors.

model {

for (i in 1:n) { # n = number of individuals

hcv[i] ~ dbern(p[i])

logit(p[i]) <- bcons + b1*age[i] + b2*age2[i] + b3*inject[i] + b4*fish[i] +

b5*transfuse[i] + b6*sex[i] + b7*dentist[i] + b8*know[i] + v[vid[i]]

}

for (k in 1:nnn) { # nnn = number of grid cells

v[k] ~ dnorm(0,tau.v)

}

## models for missing values

for (i in 1:n) {

dentist[i] ~ dbern(pd[i])

logit(pd[i]) <- bdcons + bd1*age[i] + bd2*sex[i]

}

for (i in 1:n) {

transfuse[i] ~ dbern(pt[i])

logit(pt[i]) <- btcons + bt1*age[i] + bt2*sex[i]

}

for (i in 1:n) {

fish[i] ~ dbern(pf[i])

logit(pf[i]) <- bfcons + bf1*age[i] + bf2*sex[i]

}

for (i in 1:n) {

inject[i] ~ dnegbin(pi[i], r)

log(mu[i]) <- bicons + bi1*age[i] + bi2*sex[i]

pi[i] <- r/(r + mu[i])

}

# priors

bcons ~ dnorm(0, 0.0001)

b1 ~ dnorm(0, 0.0001)

b2 ~ dnorm(0, 0.0001)

b3 ~ dnorm(0, 0.0001)

b4 ~ dnorm(0, 0.0001)

b5 ~ dnorm(0, 0.0001)

b6 ~ dnorm(0, 0.0001)

b7 ~ dnorm(0, 0.0001)

b8 ~ dnorm(0, 0.0001)

sigma2.v <- pow(sigma.v,2)

sigma.v ~ dunif(0, 100)

tau.v <- 1/sigma2.v

btcons ~ dnorm(0, 0.0001)

bt1 ~ dnorm(0, 0.0001)

bt2 ~ dnorm(0, 0.0001)

bfcons ~ dnorm(0, 0.0001)

bf1 ~ dnorm(0, 0.0001)

bf2 ~ dnorm(0, 0.0001)

bicons ~ dnorm(0, 0.0001)

bi1 ~ dnorm(0, 0.0001)

bi2 ~ dnorm(0, 0.0001)

r ~ dunif(0, 1000)

bdcons ~ dnorm(0, 0.0001)

bd1 ~ dnorm(0, 0.0001)

bd2 ~ dnorm(0, 0.0001)

}

Below is the R code to perform the logistic regression with HCV viraemia as the outcome, including estimation of missing values in the outcome.

model {

for (i in 1:n) { # n = number of individuals

hcv[i] ~ dbern(p[i])

logit(p[i]) <- bcons + b1*age[i] + b2*sex[i] + b3*nottreated[i]

}

# priors

bcons ~ dnorm(0, 0.0001)

b1 ~ dnorm(0, 0.0001)

b2 ~ dnorm(0, 0.0001)

b3 ~ dnorm(0, 0.0001)

}

**Odds ratio for the relationship between hypothesized risk factors for HCV seropositivity and age and sex**

Table A gives odds ratio estimates from study data collected in Machar Colony in March 2022.

**Table A.** Odds ratio estimates from multivariable logistic regression for the relationship between hypothesised risk factors for HCV seropositivity and age and sex using questionnaire data collected in Machar Colony in March 2022.

| **Risk factor** | **Age** | **Female** |
| --- | --- | --- |
| Number of injections received in past 12 months | 1.02 (1.01 – 1.03) | 0.71 (0.56 – 0.90) |
| Work in the fisheries sector | 1.01 (0.99 – 1.02) | 0.01 (0.00 – 0.03) |
| Receipt of a blood transfusion | 1.02 (1.01 – 1.04) | 2.56 (1.55 – 4.36) |
| Use of a dentist | 1.06 (1.04 – 1.07) | 1.00 (0.89 – 1.45) |

**Characteristics of people attending the clinic for confirmatory testing**

Table B gives the characteristics of the 162 seropositive people who did and did not attend the MSF hepatitis C clinic for confirmatory PCR.

**Table B.** Characteristics of seropositive study participants who did not attend the MSF hepatitis C clinic for confirmatory PCR testing in Machar Colony, Karachi using questionnaire data collected in March 2022.

| **Characteristic** | | **Total number (%)** | **Number (%) not attending clinic** |
| --- | --- | --- | --- |
| Gender | Female | 89 (54.9) | 16 (18.0) |
|  | Male | 73 (45.1) | 11 (15.1) |
| Age (in years) | 12-17 | 0 | - |
|  | 18-29 | 24 (14.8) | 2 (8.3) |
|  | 30-39 | 33 (20.4) | 3 (9.1) |
|  | 40-49 | 43 (26.5) | 8 (18.6) |
|  | 50-59 | 31 (19.1) | 4 (12.9) |
|  | >60 | 31 (19.1) | 10 (32.3) |
