# Supplementary material for: Prevalence and risk factors for hepatitis C virus infection in an informal settlement in Karachi, Pakistan
Source: PLOS Glob Public Health. 2023 Sep 20;3(9):e0002076. doi: 10.1371/journal.pgph.0002076 (PMC10511086; doi:10.1371/journal.pgph.0002076)
Supplement: Attachment — Submitted filename: Response to Reviewers.docx [file pgph.0002076.s005.docx]

May 27^th^, 2023

Chrispin Chaguza
Academic Editor
PLOS Global Public Health

Manuscript Title: Prevalence and risk factors for hepatitis C virus infection in an informal settlement in Karachi, Pakistan

ID: PGPH-D-23-00220

Dear Chrispin,

Many thanks for detailed review on the manuscript. We appreciate the valuable feedback that has been provided and have edited the manuscript to address each of the comments. We have also added responses to each of the comments raised by the reviewer(s) and the editors for your perusal below (in blue):

Journal Requirements:

1. Please send a completed 'Competing Interests' statement, including any COIs declared by your co-authors. If you have no competing interests to declare, please state "The authors have declared that no competing interests exist". Otherwise please declare all competing interests beginning with the statement "I have read the journal's policy and the authors of this manuscript have the following competing interests:"

Response: This has been added in the manuscript.

2. Please provide a/amend your detailed Financial Disclosure statement. This is published with the article. It must therefore be completed in full sentences and contain the exact wording you wish to be published.

Response: This has been added in the manuscript.

a. Please clarify all sources of funding (financial or material support) for your study. List the grants (with grant number) or organizations (with url) that supported your study, including funding received from your institution.

Response: The funding for this Project was received from Foundation for Innovative New Diagnostics (FIND) (website: [www.finddx.org)](http://www.finddx.org/) by Interactive Research and Development (IRD) (website:<https://ird.global/country/pakistan/>, Pakistan). The total grant amount awarded to IRD was USD 44,827/-.

b. State the initials, alongside each funding source, of each author to receive each grant.

Response: AP – FIND

c. State what role the funders took in the study. If the funders had no role in your study, please state: “The funders had no role in study design, data collection and analysis, decision to publish, or preparation of the manuscript.”

Response: This has been added in the manuscript.

d. If any authors received a salary from any of your funders, please state which authors and which funders.

Response: MM, RA, AP, received salary from the grant amount awarded to IRD by FIND.

If you did not receive any funding for this study, please simply state: “The authors received no specific funding for this work.”"

3. Some material included in your submission may be copyrighted. According to PLOS’s copyright policy, authors who use figures or other material (e.g., graphics, clipart, maps) from another author or copyright holder must demonstrate or obtain permission to publish this material under the Creative Commons Attribution 4.0 International (CC BY 4.0) License used by PLOS journals. Please closely review the details of PLOS’s copyright requirements here: PLOS Licenses and Copyright. If you need to request permissions from a copyright holder, you may use PLOS's Copyright Content Permission form.

Please respond directly to this email or email the journal office and provide any known details concerning your material's license terms and permissions required for reuse, even if you have not yet obtained copyright permissions or are unsure of your material's copyright compatibility.

Potential Copyright Issues:

Figure 1: please (a) provide a direct link to the base layer of the map (i.e., the country or region border shape) and ensure this is also included in the figure legend; and (b) provide a link to the terms of use / license information for the base layer image or shapefile. We cannot publish proprietary or copyrighted maps (e.g. Google Maps, Mapquest) and the terms of use for your map base layer must be compatible with our CC-BY 4.0 license.

Note: if you created the map in a software program like R or ArcGIS, please locate and indicate the source of the basemap shapefile onto which data has been plotted.

If your map was obtained from a copyrighted source please amend the figure so that the base map used is from an openly available source. Alternatively, please provide explicit written permission from the copyright holder granting you the right to publish the material under our CC-BY 4.0 license.

Please note that the following CC BY licenses are compatible with PLOS license: CC BY 4.0, CC BY 2.0 and CC BY 3.0, meanwhile such licenses as CC BY-ND 3.0 and others are not compatible due to additional restrictions.

If you are unsure whether you can use a map or not, please do reach out and we will be able to help you. The following websites are good examples of where you can source open access or public domain maps:
* U.S. Geological Survey (USGS) - All maps are in the public domain. ([http://www.usgs.gov](http://www.usgs.gov/))
* PlaniGlobe - All maps are published under a Creative Commons license so please cite “PlaniGlobe, [http://www.planiglobe.com](http://www.planiglobe.com/), CC BY 2.0” in the image credit after the caption. (<http://www.planiglobe.com/?lang=enl>)
* Natural Earth - All maps are public domain. ([http://www.naturalearthdata.com/about/terms-of-use](http://www.naturalearthdata.com/about/terms-of-use/)

Response: We have updated this map and the figure caption.

4. We have noticed that you have a list of Supporting Information legends in your manuscript. However, there are no corresponding files uploaded to the submission. Please upload them as separate files with the item type 'Supporting Information'.

Response: We have re-attached the files.

====================================================================

Responses to Review Comments to the Author:

Reviewer #1: The authors discuss an important topic, touching on the prevalence and risk factors for hepatitis C virus infection in Pakistan. The manuscript is well written and structured. Below are some issues the authors might want to address:

Statement on page 189 is not clear (2.5 Data Collection) “Participating household members conducted an individual questionnaire with close-ended 190 questions focusing on behaviors and exposures that have been shown to be important as risk 8 191 factors for HCV infection in other settings in Pakistan [9, 10, 26, 27].” I guess the point is that the questionnaire was administered to the participants.

Response: This has been rephrased in the manuscript.

Number of injections is one of the important independent variables in the analysis. The distribution of the number of injections received is likely to be right-skewed. Did the authors check the distribution of the number of injections to decide if treating it as categorical would make more sense?

Response: The reviewer is correct that the distribution is right skewed. We did not categorize this continuous variable to avoid loss of information in this important variable. We also consider that a linear relationship between the number of injections received in the past year and HCV seropositivity makes biological sense.

The authors could first explore the missing data mechanism (MCAR, MAR, MNAR etc) before including models that augment or account for the missing data. They could check, for example, whether the probability that the data was missing, depends on age and sex. What has been presented in Table 3 are the results from the models for the missing values if I am not mistaken.

Response: We have updated the text to clarify that we do not expect the data to be MNAR (and therefore that we consider the use of imputation valid) (Line 517-521). Since adult males in this setting are the main economically active group and were commonly less willing to give up their time (and therefore to answer all questions), we think it is likely that these data are MAR as opposed to MCAR (with age and sex major determinants). However, we did not formally test this since observing this relationship would not change how we managed missing data.

We specified separate logistic regression models to generate the missing values based on age and sex (and frequency of each variable in this setting). The output from these models is shown in Table 3. We have also further clarified this in the text (Line 262-269). We have also included the R code used in the Supplementary Materials.

Before reporting results from the full model in Table 2, did the authors investigate how the predictors were associated with the outcome on their own? The crude and adjusted odds ratios could then be reported side-by-side. It would make sense to clearly state that these were adjusted odds ratios. Is there a why reason posterior probabilities are not reported for age in Table 2?

Response: We have included unadjusted odds ratios for reference in Table 2. The methods section has also been updated. We primarily included age and sex in this model to control for confounding of the effects of the (modifiable) risk factors in this setting, which was the reason for not including posterior probabilities. This has been clarified in Line 256-258.

Figure 3 is not clear. Please consider increasing the resolution and making the labels on the x-axis clearer.

Response: We have improved Figure 3

Reviewer #2: This paper presents the results of a serosurvey of hepatitis C conducted in a slum in Karachi.

The sampling framework is well described and appropriate.

Comments:
1. I would not consider the inclusion of the finding of the association with working in fisheries in the abstract since this association has wide confidence intervals substantially overlapping a null effect.

Response: This change has been made in the manuscript.

2. "The seroprevalence was more than double the reported seroprevalence in the Sindh Province."
Should include "previously reported"

Response: This change has been made in the manuscript.

3. This section is redundant and can be removed: "People who are HCV seropositive may fall into three categories: 1. Infected with HCV in the past but naturally cleared the infection; 2. Infected in the past and treated and cured; 3. Currently HCV infected."

Response: This change has been made in the manuscript.

4. "Individuals found to have hepatitis C infection were managed as patients following standard MSF clinical protocols." It would be informative to briefly describe what therapy was provided.

Response: These details have been added in the manuscript (Line 182-190).

5. I am uncertain how the Bernoulli trial method for estimating viraemic prevalence has contributed to the validity of the viraemic prevalence estimates. A multiple imputation method would be more likely to be informative. An analysis of the known characteristics of participants who did, and did not attend for confirmatory testing will be useful to ascertain whether bias is likely to affect viraemic estimates.

Response: The Bernoulli method was simple mean imputation (with the mean provided by the viraemic ratio). We have added some additional analysis of the characteristics of HCV viraemia among seropositive people (Section 2.6.3). However, given the small available sample size of seropositive people, and a limited set of characteristics in our data that would be expected to influence viral clearance, and therefore viraemia (as opposed to HCV exposure), we have limited this analysis to age, sex, and treatment history. This additional analysis has allowed us to predict HCV viraemic status where this was missing, and we have therefore removed the Bernoulli estimation procedure.

6. A random effects variable was included at the grid level but not household level? A multilevel model should account for household level clustering, since transmission within the household might be expected.

Response: We have included evaluation of the contribution of different grouping levels (household, grid, and grid and household) as random effects to model fit (Line 245-249). The final model remains the same as the inclusion of a random effect at the household level does not improve predictive error (Line 355-357).

7. The imputation of missing data for the multivariable logistic regression has not been fully described. What assumptions were made about missingness and how much missing data was imputed? Please describe this in a supplementary appendix.

Response: We have added some additional information on the expected missingness mechanism (Line 517-521). The amount of missingness present in each variable is given in Table 1, and this has been clarified in the text (Line 320). We have also included the R code used to estimate these models in the supplementary materials.

8. The selection process as described is a two-stage sampling method with grid- and household level sampling. Prevalence should be adjusted by sampling weights accounting for the two-stage process rather than being treated as a simple random sampling in the analysis. I cannot see any mention of survey weights. These should be incorporated into all models including prevalence estimation and regression.

Response: We note that only households were “sampled” – almost all grid cells were included (with a small number of small “edge” grid cells excluded). This additional level was included for convenience and since it provides a useful contextual level for exploring spatial clustering. We also aimed to include all people in selected households for sampling. Our sample (at least in design) therefore comes close to being a simple random sample. However, since not all household members agreed to participate when it came to sampling, we have updated the prevalence estimates to account for clustering at the level as well as a household level sampling weight – see additions to methods and results.

Given the points above about the closeness of our sample to a simple random sample, we have not included sampling weights in the regression analysis.

9. The table 1 baseline characteristics should include age summary statistics.

Response: This has been added.

Thank you once again for your time and consideration. I look forward to hearing from you.

Sincerely,

Munazza Mansoor
