# Supplementary material for: Prevalence and risk factors for hepatitis C virus infection in an informal settlement in Karachi, Pakistan
Source: PLOS Glob Public Health. 2023 Sep 20;3(9):e0002076. doi: 10.1371/journal.pgph.0002076 (PMC10511086; doi:10.1371/journal.pgph.0002076)
Supplement: Attachment — Submitted filename: Response to Reviewers.docx [file pgph.0002076.s006.docx]

August 16^th^, 2023

Chrispin Chaguza
Academic Editor
PLOS Global Public Health

Manuscript Title: Prevalence and risk factors for hepatitis C virus infection in an informal settlement in Karachi, Pakistan

ID: PGPH-D-23-00220

Dear Chrispin,

Many thanks for detailed review on the manuscript. We appreciate the valuable feedback that has been provided and have edited the manuscript to address all comments. We have also added responses to each of the comments raised by the reviewer(s) and the editors for your perusal below (in blue):

Journal Requirements:

1. Please review your reference list to ensure that it is complete and correct. If you have cited papers that have been retracted, please include the rationale for doing so in the manuscript text, or remove these references and replace them with relevant current references. Any changes to the reference list should be mentioned in the rebuttal letter that accompanies your revised manuscript. If you need to cite a retracted article, indicate the article’s retracted status in the References list and also include a citation and full reference for the retraction notice.

Response: The reference list has been updated and the following reference has been removed: Ministry of National Health Services. Guidelines for the treatment of the persons with chronic hepatitis C infection, Pakistan. 2020. Government of Pakistan, Ministry of National Health Services, Regulations and Coordination.

2. Please amend your detailed Financial Disclosure statement. This is published with the article. It must therefore be completed in full sentences and contain the exact wording you wish to be published.

Response: The statement has been amended.

a. State the initials, alongside each funding source, of each author to receive each grant.

Response: The funding for this Project was received from Foundation for Innovative New Diagnostics (FIND) (website: [www.finddx.org)](http://www.finddx.org/) by Interactive Research and Development (IRD) (website:<https://ird.global/country/pakistan/>). The grant was awarded to AP.

Médecins Sans Frontières (MSF) funding is predominantly based on individual donating small amounts – 80% of its financial resources are allocated to fulfilling social missions and remaining 20% on general management and fundraising costs. Diagnostic testing and treatment for participants in the Project were funded under MSF operational budgets. MSF funds are tightly controlled and the audited financial reports are publicly available (website:<https://www.msf.org/reports-and-finances>).

b. If any authors received a salary from any of your funders, please state which authors and which funders.

Response: Through the funding allocated by FIND to IRD, AP, MM, RA received a salary. Whereas, the diagnostic testing and treatment for HCV positive patients were managed under MSF operational budgets mentioned above, with authors WDG, KA and MA receiving a salary from MSF during the Project and clinical management tenure.

If you did not receive any funding for this study, please simply state: “The authors received no specific funding for this work.”

**Review Comments to the Author**
Reviewer #1: All the comments I made have been addressed. Table 1 could be formatted better. For example, the authors present Age (in years) (1300 (100%)) to show the number of responses and percentage of the total. The (100%) could be removed and a footer could be used to indicate the N for variables that had missing responses from some participants.

Response: We have removed the percentages from the table but feel including the N after each characteristic is the clearest way to present this missingness.

Reviewer #2: The changes made in this revised article have improved the manuscript.

I have a few additional comments:

1. I would update table 1 to also include viraemic prevalence (with the proportion and CIs adjusted for available data).

Response: We have added a new table (Table 4) that contains viraemic prevalence by age and gender. Since the risk/protective factors shown in Table 1 are considered to influence exposure (and therefore seropositivity) rather than infection (and therefore viraemia) we have not summarized viraemic prevalence by these variables.

2. Table 3 presents evidence of the association between indicator variables of sex and age vs other regression variables. As it does not report an relationship with HCV prevalence I would remove this table to the appendix, as it is may be easily misinterpreted as referring to HCV.

Response: This has been moved to the Supplementary Materials attached as part of the Manuscript under Table S1.

3. The authors have not reported participant characteristics data stratified by present/missing HCV RNA result in a supplementary table as requested in the first review. This remains an important step to identify response bias and I recommend it.

Response: We have added these details to the supplementary materials focusing on age and sex (since we do not expect other variables would influence viraemia in seropositive people). We do not formally evaluate these associations but note that these variables are included in the regression that is used to estimate missing HCV viraemia values.

4. Could figure 4 be accompanied with any additional spatial information/satellite imaging which might inform the observed clustering? Could figure 4 show any relevant spatial clustering statistics.

Response: We agree a detailed examination of the spatial distribution of HCV seropositivity in Machar Colony would be valuable, but we are not aware of spatially explicit data sets that would support this. In the discussion, we describe some possible reasons for the observed spatial clustering. A visual examination of satellite imagery for Machar Colony does not inform the observed clustering. We removed satellite imagery from the previous version of Figure 1 due to journal licensing requirements. We have added the relevant spatial clustering statistics (from the spatial scan statistic) to the caption for Figure 4.

5. Could figure 2 also be stratified by sex (eg. male/female estimates for each age group)? Also the x-axis is categorical it should therefore include the proper categorical labelling eg. "15-19" and not "15". Better yet, use of a spline for age with 95% CI or CrI would show the continuous relationship overall, and stratified by sex.

Response: This figure has been updated following these recommendations.
